# Topological phase transition in quasi-one dimensional organic conductors

**DOI:** 10.1038/srep17358

**Published:** 2015-11-27

**Authors:** Xiao-Shan Ye, Yong-Jun Liu, Xiang-Hua Zeng, Guoqing Wu

**Affiliations:** 1 College of Physics Science and Technology, Yangzhou University, Yangzhou 225002, China; 2 Department of Physics and Astronomy, University of California, Los Angeles, California 90095, USA

## Abstract

We explore topological phase transition, which involves the energy spectra of field-induced spin-density-wave (FISDW) states in quasi-one dimensional (Q1D) organic conductors, using an extended Su-Schrieffer-Heeger (SSH) model. We show that, in presence of half magnetic-flux FISDW state, the system exhibits topologically nontrivial phases, which can be characterized by a nonzero Chern number. The nontrivial evolution of the bulk bands with chemical potential in a topological phase transition is discussed. We show that the system can have a similar phase diagram which is discussed in the Haldane’s model. We suggest that the topological feature should be tested experimentally in this organic system. These studies enrich the theoretical research on topologically nontrivial phases in the Q1D lattice system as compared to the Haldane topological phase appearing in the two-dimensional lattices.

Topological superconductors and topological insulators have been of considerable interest in condensed matter physics because of their nontrivial topological properties as new states of quantum matter and potential remarkable technological applications. Unfortunately these materials are relatively rare among condensed matter materials and understanding their properties has huge challenges due to the complexity of their topological states^1^,^2^,^3^,^4^,^5^,^6^,^7^,^8^,^9^,^10^. It is believed that topological superconductors have a full pairing gap in the bulk and gapless surface states which are usually related to Majorana fermions^11^,^12^, while in close analogy to the topological superconductors the topological insulators can be characterized by a full insulating gap in the bulk and gapless edge or surface states protected by time-reversal symmetry. Therefore, different edge states have different properties. Moreover, an applied high magnetic field can break a time-reversal-invariant symmetry, leading to a chiral edge state and a quantized Hall effect^13^. In contrast, a spin-orbit coupling can preserve the time-reversal-invariant symmetry and produce a helical edge structure^6^, as well as quantum spin Hall effect which has a quantized spin-Hall conductance and a vanishing charge-Hall conductance. Thus the quantized Hall effect and the quantum spin Hall effect have different origins and characters.

Various models have been proposed for the understanding of the topological states and the topological properties. For example, the existence of a quantum spin Hall state was explained by the Kane and Mele lattice model^6^, which corresponds to two copies of the Haldane model developed earlier for the graphene material^14^. According to the Kane and Mele model, the spin up electron exhibits a chiral integer quantum Hall Effect while the spin down electron exhibits an anti-chiral integer quantum Hall effect. This gives a zero charge-Hall conductance and a fixed quantum value of spin-Hall conductance. Soon after, the existence of spin-orbit coupling was understood as a momentum-dependent magnetic field coupling to the spin of the electron, using the model developed by Bernevig and Zhang^15^. An important view is that all these models indicate that the time-reversal symmetry plays a fundamental role in guaranteeing the topological stability of the states and in the topological properties. On the other hand, some researchers suggest the possibility of a cold atom crystal fabrication with the use of artificial optical lattice^16^,^17^, considering the engineering difficulty of applying the Haldane model which requires the applications of an upward magnetic field for spin-up electrons and an downward magnetic field for spin-down electrons at meanwhile.

Remarkably, recent progress in understanding the topological properties have led to the experimental observation of the topological states (including those in the HgTe quantum wells) and the discovery of new time-reversal-invariant topological materials, including Be_2_Te_3_, Be_2_Se_3_ and Cu_*x*_Be_2_Se_3_^12^.

The Haldane model gives the conceptual basis for theoretical research on topological insulators. To enrich theoretical research, in this work, we put forward a novel approach to study the Haldane model’s phase diagram in Q1D systems. Unlike the general schemes which concentrate on two dimensional systems by introducing a complex next-nearest-neighbor (NNN) hopping term while without any net magnetic flux through a unit cell of the honeycomb lattice, we will explore the realization of Haldane’s phase diagram in Q1D organic systems with special modulated order parameters. We consider FISDW states in the Q1D lattice using a tight-binding model, which includes the dimerization effect. We demonstrate that nontrivial edge states emerge with the introduction of the dimerization effect when the FISDW order parameter is changed. If we define a parameter to describe the dimerization effect, we can use this parameter and momentum to define a topological invariant in an extended two dimension parameter space to distinguish different topological phases. When we change the system filling, the topological invariant will give rise to a phase diagram similar to that of the two dimension Haldane’s model. The topologically nontrivial edge states in our model may be observable in this system because the FISDW order and the dimerization effect in the Q1D organic conductors (TMTSF)_2_*X* have been discovered by experiments.

## Results

(TMTSF)_2_*X* are the Q1D organic compounds that have parallel conducting chains in the *a* − *b*-plane of the crystal lattice, where TMTSF is the abbreviation of tetramethyltetraselenafulvalene and *X* represents an inorganic anion (*X* = PF_4_ and ClO_4_). (TMTSF)_2_*X* are charge-transfer salts that have unpaired electrons residing in the Se *π*-orbitals (*π*-electrons) of the donor unit TMTSF^18^,^19^,^20^. The *π*-electrons can be delocalized throughout the crystal and give rise to the metallic conductivity with a Q1D conducting band, as a result of the inter-molecules *π*-orbital overlaps along the conducting chains (stacking axis of the planar-shape TMTSF molecules along the crystal a-axis)^20^,^21^,^22^. (TMTSF)_2_*X* exhibit interesting behaviors when a strong magnetic field is applied perpendicular to the *a* − *b*-plane), with which a metal-insulator phase transition appears at low temperatures. The phase transition is known to be caused by FISDW, a long-range spin ordering effect due to the application of the externally applied magnetic field^19^,^20^,^21^,^22^,^23^. As the applied magnetic field is increased further, a series of quantum phase transitions within the FISDW states occurs^22^,^23^,^24^. Here we discuss the FISDW state with the energy spectrum of the (TMTSF)_2_*X* electron system. According to the tight-bonding model, (TMTSF)_2_*X* are characterized by a simple electron spectrum: 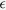
(**k**_*x*_, **k**_*y*_) = 2*t*_*a*_ cos**k**_*x*_ + 2*t*_*b*_ cos**k**_*y*_ where *t*_*a*_/*t*_*b*_ = 10. Each quantized FISDW is characterized by an order parameter 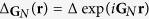, where *N* is an integer^18^,^23^. Here Δ characterizes a change of the strength of the order parameter, and *G*_*N*_ = (*N***G**, *π*/*b*), where *b* is the lattice constant along the *b*-axis (also the *y*-axis direction), and **G** = *ebH*/*ħc* (*e* is the electron charge, *H* is the magnitude of the applied magnetic field, *ħ* is the Planck constant, and *c* is the velocity of light). The applied magnetic field **H** is applied ⊥ to the conducting chain of the Q1D compounds, i.e., **H** = (0, 0, *H*). Thus we also have *a***G** = 2*πϕ*/*ϕ*_0_, where *a* is the lattice constant along the crystal *a*-axis, *ϕ* = *abH* is the magnetic flux per unit cell, the *ϕ*_0_ = *hc*/*e* is the magnetic flux quantum. By considering a charge particle hopping between nearest neighbors with hopping amplitude *t* in the presence of FISDW, we obtain the Hamiltonian: 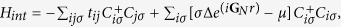 where *μ* is the chemical potential.

**Figure 1:**
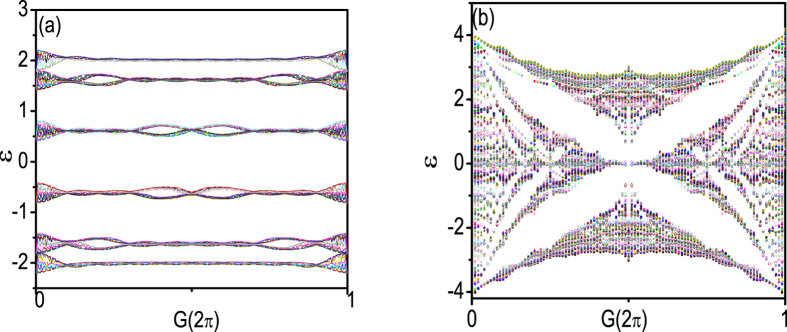
Energy varying with the wave vector G of the FISDW for (TMTSF)_2_X with *t*_*a*_/*t*_*b*_ = 10 and the FISDW strength Δ = 0.2 (**a**), Δ = 2 (**b**), here *t*_*a*_ = 1 is set as the unit of energy^25^.

For simplicity, first, we will assume that the FISDW potential is spin independent. Then we will discuss the spin-dependent case. In the absence of the FISDW, the organic compounds of the (TMTSF)_2_*X* are metals. When a strong magnetic field is applied ⊥ to the conducting chain of the Q1D compounds, there is a phase transition from the metallic state to a FISDW state. In Fig. 1(a), we show the band structure of it in presence of the FISDW with a small FISDW strength (Δ = 0.2, here *t*_*a*_ = 1 is set as the unit of energy). Each band is broad and there are two sine or cosine modulated forms in it. The bands broaden are bigger at **G** = (2*π*/5)*n(*n* = 0, 1, 2, 3, 4). There are energy gaps near these points in each broaden band. That is, each band is split into two sub-bands due to the FISDW order parameter. We call one conductanc sub-band and the other valence sub-band. With increasing of the strength of the FISDW (Δ = 0.6), the energy bands begin to touch each other. The energy gaps near commensurate wave vector points become larger. When Δ = 2, all these energy gap edges in each band touch each other. The energy spectrum with different **G** is presented in Fig. 1(b). We can find that it has a similar Hofstadter butterfly energy spectrum structure of the two-dimensional lattice system^25^.

**Figure 2:**
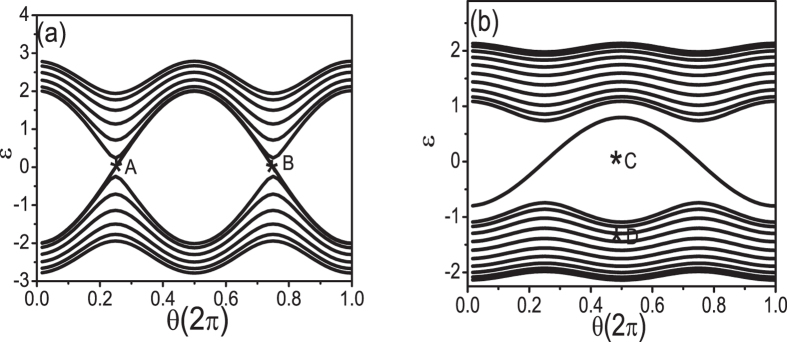
Under open boundary conditions, energy varies with the dimerization effect induced **θ** for (TMTSF)_2_*X* materials with *t*_*a*_/*t*_*b*_ = 10 and the FISDW strength Δ = 2, the dimerization strength *δt* = 0.1 (**a**), the FISDW strength Δ = 0.4, and the dimerization strength *δt* = 0.3(**b**).

Owning to soft properties, there are strong electron-phonon couplings in these organic conductors. Here, we discuss the dimerization effect on the FISDW order using the SSH model. The SSH model is a standard tight-binding model with spontaneous dimerization proposed by Su, Schrieffer and Heeger to describe the Q1D system^24^. Considering the dimerization effect, we obtain the following Hamiltonian:


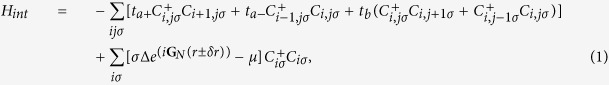


where *t*_*a*+_ = *t*_*a*_ + *δt* and *t*_*a*−_ = *t*_*a*_ − *δt* (*δt* representing the dimerization strength). The FISDW order parameter 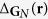 equals to Δexp(*i***G**_*N*_**(r** + **δr)**) at odd sites and Δexp(*i***G**_*N*_**(r** − **δr)**) at even sites along the chain. We set **G** ⋅ *δr* = *θ* in the following discussions. From the FISDW order parameter, we can see that the energy spectrum varies with the vector **G**. When **G** = 2*πϕ*/*ϕ*_0_ = *π*, the energy spectrum consists of two Dirac cones(Fig. 2(a)). These two cones are separated by a gap when we consider the dimerization effect.

**Figure 3:**
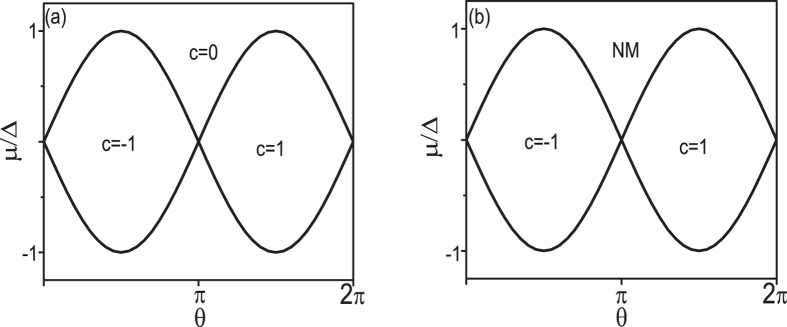
Phase diagram for the system with different chemical potential when the FISDW strength Δ = 0.4, and the dimerization strength *δt* = 0.3. (**a**) the FISDW strength Δ = 2, and the dimerization strength *δt* = 0.1(**b**). *C* is the chern number. The region with the Chern number *C* = 0 is the trivial insulator phase, *C* = 1 and *C* = −1 are corresponding topological nontrivial insulator phases, respectively. NM is the normal metal phase.

**Figure 4:**
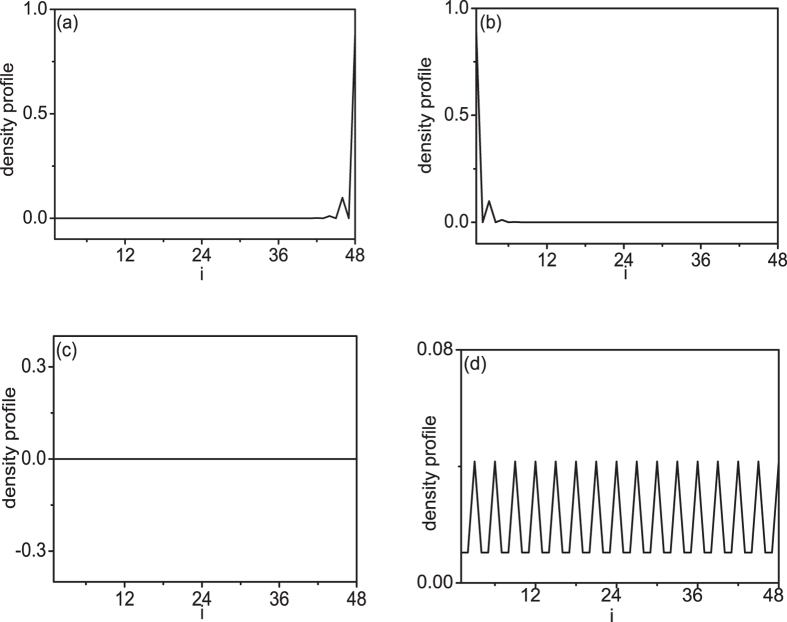
Density profiles of the system in different states labeled by the capital letter inFig. 2, which are localized at the edge state A(*θ* = *π*/2) (a), the edge state B(*θ* = 3*π*/2) (b), the insulation state C (c), and the metal state D (d). Here, we choose *N* = *L* × *L* = 48 × 48.

Now, we put the system under open boundary conditions (OBC). As showed in Fig. 2(a), there are edge states in the gap. Generally, the edge states are associated with the nontrivial topological properties of the systems. If we continuously change the parameter *θ*, a topological phase transition occurs at *θ* = *π*/2 or 3*π*/2. Thus, we can define a Chern number *C* in the (*k, θ*) parameter space to describe the topological phase, 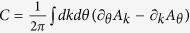, where *A*_*k*_ = *i *< *φ*(*k*)|∂_*k*_|*φ*(*k*) > and *A*_*θ*_ = *i* < *φ*(*k, θ*)|∂_*θ*_|*φ*(*k, θ*)> with *φ* to be the occupied Bloch state. We find that *C* = −1 when *θ* ∈ (−*π*/2, *π*/2), and *C* = 1 when *θ* ∈ (*π*/2, 3*π*/2). A phase transition occurs at *θ* = *π*/2 and *θ* = 3*π*/2. The Chern number is well defined when the gap of the bulk states is fully opened. This phase transition is relevant to the FIDSW strength Δ and the dimerization strength *δt*. We also find that *C* = 0 with different Δ and *δt*. In Fig. 3, we display the phase diagram in the parameter space of *θ* and chemical potential *μ* at *δt*/Δ = 0.4/0.3. We find that when Δ < 0.5 and *δt*/Δ ~ 1, the phase diagram is always similar to that shown in Fig. 3(a) in the parameter space *θ* and chemical potential *μ*. We can explain qualitatively the origin of the phase transition. As shown in Fig. 2, there exist edge states in the half magnetic-flux FISDW state for the Q1D systems. The existence of the edge states can be examined through the LDOS spectra. In Lehmann representation at zero temperature: 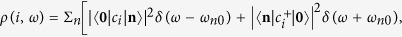 where *ω*_*n*0_ = *E*_*n*_ − *E*_0_, and 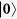 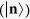 is the ground state with energy *E*_0_ (the *n* − *th* excited state with energy *E*_*n*_). As the LDOS *ρ*(*i*,*ω*) is proportional to the density of state at site *i*, in Fig. 4, we show the density of state at different sites along the chain(*a*) direction (we make an average result in *b* direction). We can see that the edge state is localized at one side of the system as the dimerization effect gives *θ* = *π*/2 (Fig. 4(a)), and transfers to the other side of the system as *θ* equals 3*π*/2 (Fig. 4(b)). The energy of this edge state changes with the dimerization effect in the form *E*_0_ cos*θ* (*E*_0_ is the biggest excited state energy). This can be seen clearly in Fig. 2(b). If the dimerization effect induced gap is larger than *E*_0_, the density of state at different sites is zero except the edge site. In this case, the system is in topological insulator state. If the dimerization effect induced gap is not big enough, the density of state at different sites is shown in Fig. 4(d) when energy is greater than *E*_0_. In this case, the system will go into a metal state when energy is greater than *E*_0_. The LDOS can be directly measured experimentally by scanning tunnelling microscope experiments. So the above numerical results for LDOS may be a good suggestion to experimental observations. Some researchers suggested one use LDOS to detect zero mode in topological superconductors^26^. Here, we suggest the topological phase should be tested experimentally using LDOS to detect the edge state.

## Discussions

To understand the phase diagram, we also give a connection of the Q1D model to the Haldane model. We make a Fourier transformation on Eq.(1). Then the Hamiltonian can be written in the form of


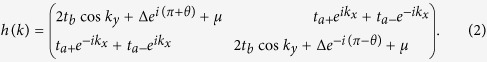


This *h*(*k*) gives a band energy gap 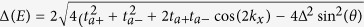. It is easy to find the phase boundary given by 2*δt*/Δ = sin*θ*, corresponding to the close of the energy gap for *k*_*x*_ = *π*/2 or *k*_*x*_ = 3*π*/2. Around the phase boundary, one can numerically calculate the Chern number. The numerical calculations show that the phase diagram has a similar structure to that appears in the Haldane model. In fact, when we review the Haldane model, we will find that our system has a similar Hamiltonian to Haldane’s. For the convenience of comparison, we briefly review the Haldane model. The Haldane model Hamiltonian is given by 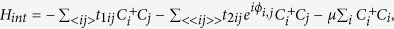 where the summation ∑_*i*_ is defined in the 2*D* honeycomb lattice, which is composed of two sublattices, *t*_1_ denotes the nearest-neighbor hopping amplitude and *t*_2_ denotes the next nearest-neighbor hopping amplitude. A complex phase *ϕ*_*ij*_ is introduced to the next nearest neighbor hopping *t*_2_. The magnitude of the phase is set to be |*ϕ*_*ij*_| = *ϕ*, and the direction of the positive phase is anticlockwise (Note the net flux is zero in one unit cell). The Haldane model Hamiltonian can be written in the momentum space as:


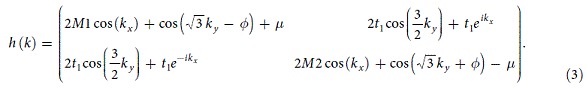


where 
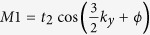
 and 
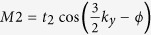
. In comparison Eq. (2) with Eq. (3), if we take the real part Δ*e*^*i*(*π*±*θ*)^ = cos(*π* ± *θ*), the form of this Hamiltonian is very similar to that in Eq. (2). This comparison gives us a straightforward understanding of the connection of the phase diagram of our model with which in the well-known Haldane model. For a spin-dependent system, we take *c*_*i*_ = (*c*_*i*↑_, *c*_*i*↓_) for spin-up and spin-down fermionic operators. Thus, the Hamiltonian in Eq. (1) can be extended to the spin-dependent one. From the same procedure as the spinless case to simulate the topology, the spin-dependent Chern number can be also defined in the (*k, θ*)-space under PBCs as 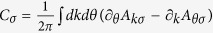 (*σ* = ↑ or ↓), where 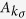 and *A*_*θσ*_ are the spin-dependent Berry connection. Using these spin-dependent Chern numbers, a total topological invariant is constructed as *ν* = (*C*_↑_ − *C*_↓_)/2, which demonstrates the *Z*_2_-type topology. We can see that as long as the bulk gap does not close, the topology of the system will not change.

We notice that (TMTSF)_2_*X* materials exhibit a sequence of phase transitions between different FISDWs when the magnetic field is increased further^18^,^19^,^20^,^21^. Within each FISDW phase, the value of the Hall resistance remains constant, that is, the quantum Hall effect is observed. Considering these experimental observations, we think the above phase diagram may be observed in this Q1D system. However, the above nontrivial topological properties of the systems depend on the FISDW vector **G**. When **G** is different from *π*, the system goes into metal states or a trivial insulator. We also notice that the FISDW vector **G** is determined by the applied magnetic field. Thus, we think the half magnetic-flux FISDW state can appear in this system when the magnetic field is controlled. In the real Q1D organic conductors (TMTSF)_2_*X*, the dimerization strength *δt* is far smaller than the FIDSW strength Δ. Considering this condition, the phase diagram shown in Fig. 3(b) may appear.

## Summary

In summary, we explore the extended SSH model structure energy spectrum of the Q1D organic conductors subject to the FISDW state. We find that energy spectrum can exhibit topologically nontrivial phases when the system is in the half magnetic-flux FISDW state. The topologically nontrivial phases due to FISDW and the dimerization effect are also investigated theoretically. We find that the topologically nontrivial phases can be characterized by a nonzero Chern number. The nontrivial evolution of the bulk bands with the dimerization in the topological phase transition is discussed. We find that the system shows a similar phase diagram to that using the Haldane’s model. These studies enrich theoretical research on Haldane topological phase in the Q1D lattice system in comparison with that in the two-dimensional lattices.

## Additional Information

**How to cite this article**: Ye, X.-S. *et al.* Topological phase transition in quasi-one dimensional organic conductors. *Sci. Rep.* **5**, 17358; doi: 10.1038/srep17358 (2015).
